# Deciding the timing of aneurysm coiling and obstetric delivery in pregnant patients presenting with ruptured intracranial aneurysms: a case report and review

**DOI:** 10.1093/bjrcr/uaag012

**Published:** 2026-04-11

**Authors:** Aly Shaaban, Abhishekh Hulegar Ashok, Robin J Borchert, Cinzia Cammarano, Lucy Miller, Basil Nourallah, Tamara Tajsic, Matthew Guilfoyle, Alison Wilson, Richard Haddon, Aoife Quinn, Nathan Chan

**Affiliations:** Department of Radiology, University of Cambridge School of Clinical Medicine, Cambridge, CB2 0QQ, United Kingdom; Department of Radiology, University of Cambridge School of Clinical Medicine, Cambridge, CB2 0QQ, United Kingdom; Department of Interventional Neuroradiology, Cambridge University Hospitals NHS Foundation Trust, Cambridge, CB2 0QQ, United Kingdom; Department of Radiology, University of Cambridge School of Clinical Medicine, Cambridge, CB2 0QQ, United Kingdom; Department of Interventional Neuroradiology, Cambridge University Hospitals NHS Foundation Trust, Cambridge, CB2 0QQ, United Kingdom; Department of Anaesthesia, Cambridge University Hospitals NHS Foundation Trust, Cambridge, CB2 0QQ, United Kingdom; Department of Anaesthesia, Cambridge University Hospitals NHS Foundation Trust, Cambridge, CB2 0QQ, United Kingdom; Department of Anaesthesia, Cambridge University Hospitals NHS Foundation Trust, Cambridge, CB2 0QQ, United Kingdom; Department of Neurosurgery, Cambridge University Hospitals NHS Foundation Trust, Cambridge, CB2 0QQ, United Kingdom; Department of Neurosurgery, Cambridge University Hospitals NHS Foundation Trust, Cambridge, CB2 0QQ, United Kingdom; Department of Obstetrics, Cambridge University Hospitals NHS Foundation Trust, Cambridge, CB2 0QQ, United Kingdom; Department of Anaesthesia, Cambridge University Hospitals NHS Foundation Trust, Cambridge, CB2 0QQ, United Kingdom; Neurosciences and trauma critical care unit, Cambridge University Hospitals NHS Foundation Trust, Cambridge, CB2 0QQ, United Kingdom; Department of Interventional Neuroradiology, Cambridge University Hospitals NHS Foundation Trust, Cambridge, CB2 0QQ, United Kingdom

**Keywords:** Aneurysm, Coil embolisation, pregnancy, caesarean section

## Abstract

The optimal treatment strategy for intracranial aneurysms (IA) in pregnancy is unclear, particularly in the third trimester of pregnancy and during the intrapartum period. We discuss a case of a ruptured IA at 39 + 3 weeks of gestation, during prelabour and review literature focusing on the order of treatment and delivery. A 4 × 6 × 3 mm aneurysm arising from the left posterior communicating segment of the internal carotid artery was embolised with multiple detachable coils, followed by a caesarean section (CS) with good maternal and foetal outcomes. Our review of the literature identified 29 reported cases of ruptured intracranial aneurysms in pregnancy. After 22 weeks of gestation, mortality was higher amongst patients who delivered first (3/9) compared to those who were coiled first (1/11). Our case and the published literature support the approach of securing the aneurysm before delivery to improve maternal outcomes.

## Introduction

Managing subarachnoid haemorrhage (SAH) caused by an intracranial aneurysm in late pregnancy poses distinct challenges, requiring careful attention to both maternal and foetal health. Near-term and during labour, decision-making surrounding management must balance the risks of delivering the foetus first versus securing the aneurysm beforehand. Currently, there are no guidelines specifying the ideal treatment approach for these patients, particularly during late gestation.^1^ Our case represents the latest gestation age at which coiling is performed before delivery.

Managing these cases requires input from the multidisciplinary team and the patient. As delivery of the foetus during early pregnancy poses significant challenges to foetal survival,^2^ the consensus is to treat the aneurysm following standard guidelines,^3^ whilst making reasonable modifications to protect the foetus.^4^ There is no consensus on the treatment order in patients with ruptured IAs who present in late pregnancy, although endovascular techniques for securing aneurysms are often preferred over clipping due to lower morbidity and mortality.^3^ Some authors suggested securing the aneurysm first to prevent re-bleeding during labour,^5^ whilst others favour delivery first to allow optimum conditions to secure the aneurysm.^4^

We describe a case where a pregnant woman in her twenties presented with a ruptured IA in the third trimester during prelabour. We also performed a systematic review of the literature, looking at the management strategies for ruptured intracranial aneurysms during pregnancy, focusing on the sequence of treatment and delivery. By analysing 28 cases with defined treatment orders, we offer practical, clinically relevant insights that support prioritising aneurysm treatment prior to delivery and present the latest known case of successful coiling before delivery at term, providing insight into a clinical field with limited consensus.

## Case presentation

**Figure 1:**
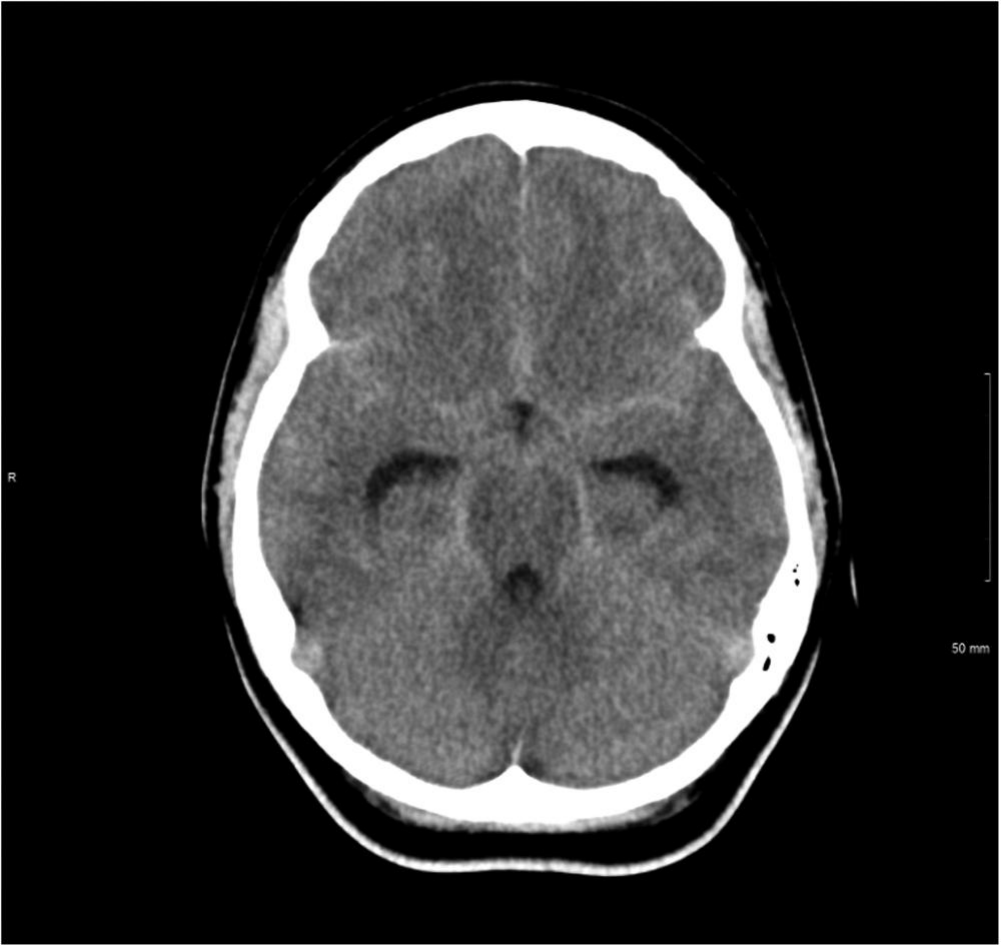
CT head at presentation demonstrating diffuse SAH with dilatation of the temporal horns indicating acute hydrocephalus.

Written informed consent was obtained from the patient for publication of this case review, including accompanying images. A primigravida in her twenties presented at 39 + 3 weeks of gestation with a severe, sudden-onset headache. She had a Glasgow Coma Scale (GCS) of 15 and no neurological deficits at presentation. Initial CT head imaging showed extensive subarachnoid haemorrhage in the basal cisterns, bilateral sylvian fissures, intra-hemispheric fissures and bilateral frontal sulci (Figure 1). CT angiography revealed an irregular, narrow-necked aneurysm measuring 3 × 3 × 8 mm in the left posterior communicating segment of the internal carotid artery. Furthermore, she had 1 episode of hypertension at 150/88 and 1+ protein in her urine on admission. Her blood pressure decreased to an average of 140/80, and trace proteinuria over the following 12 hours without medical management. Before this presentation, her pregnancy was uncomplicated with no significant past medical history. She was a long-term smoker of 5-7 cigarettes a day without a history of hypertension. She had stopped smoking 2 months into her pregnancy.

## Intervention

Following discussions involving the patient, neuro-interventional radiology, neurosurgery, neuro-anaesthesia, neurocritical care, obstetrics, obstetric anaesthesia and neonatal intensive care, it was decided to proceed with endovascular coiling followed by a lower-segment CS under the same general anaesthetic.

**Figure 2:**
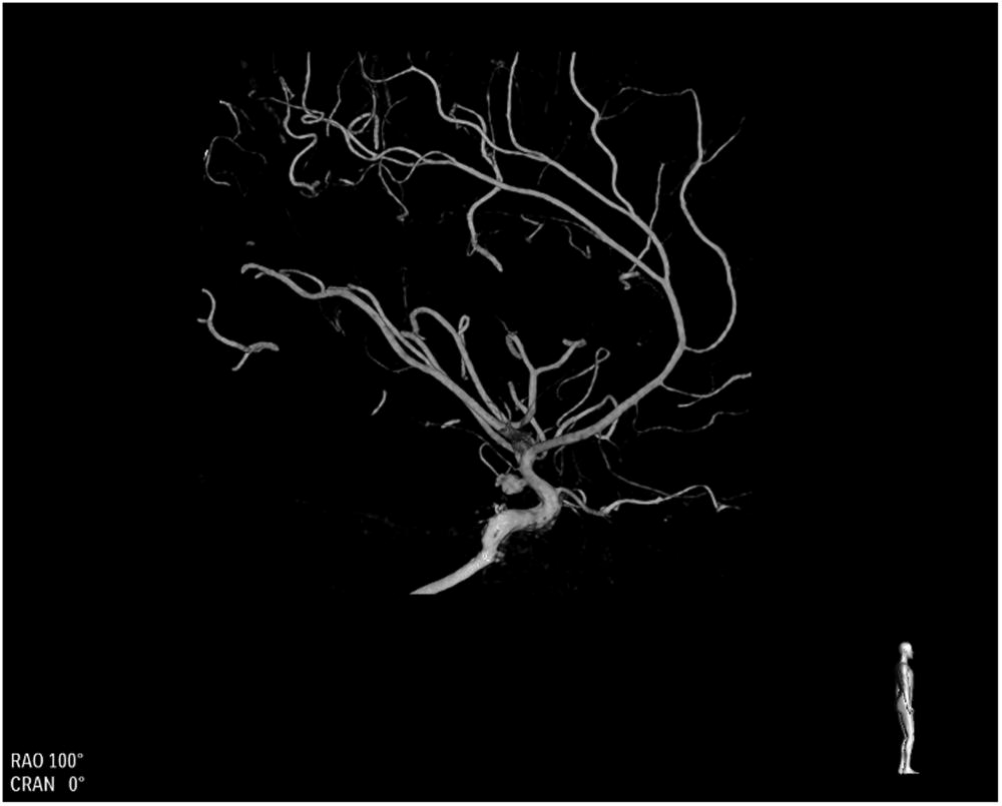
3D rotational angiography demonstrating a narrow-necked left posterior communicating segment internal carotid artery aneurysm.

**Figure 3:**
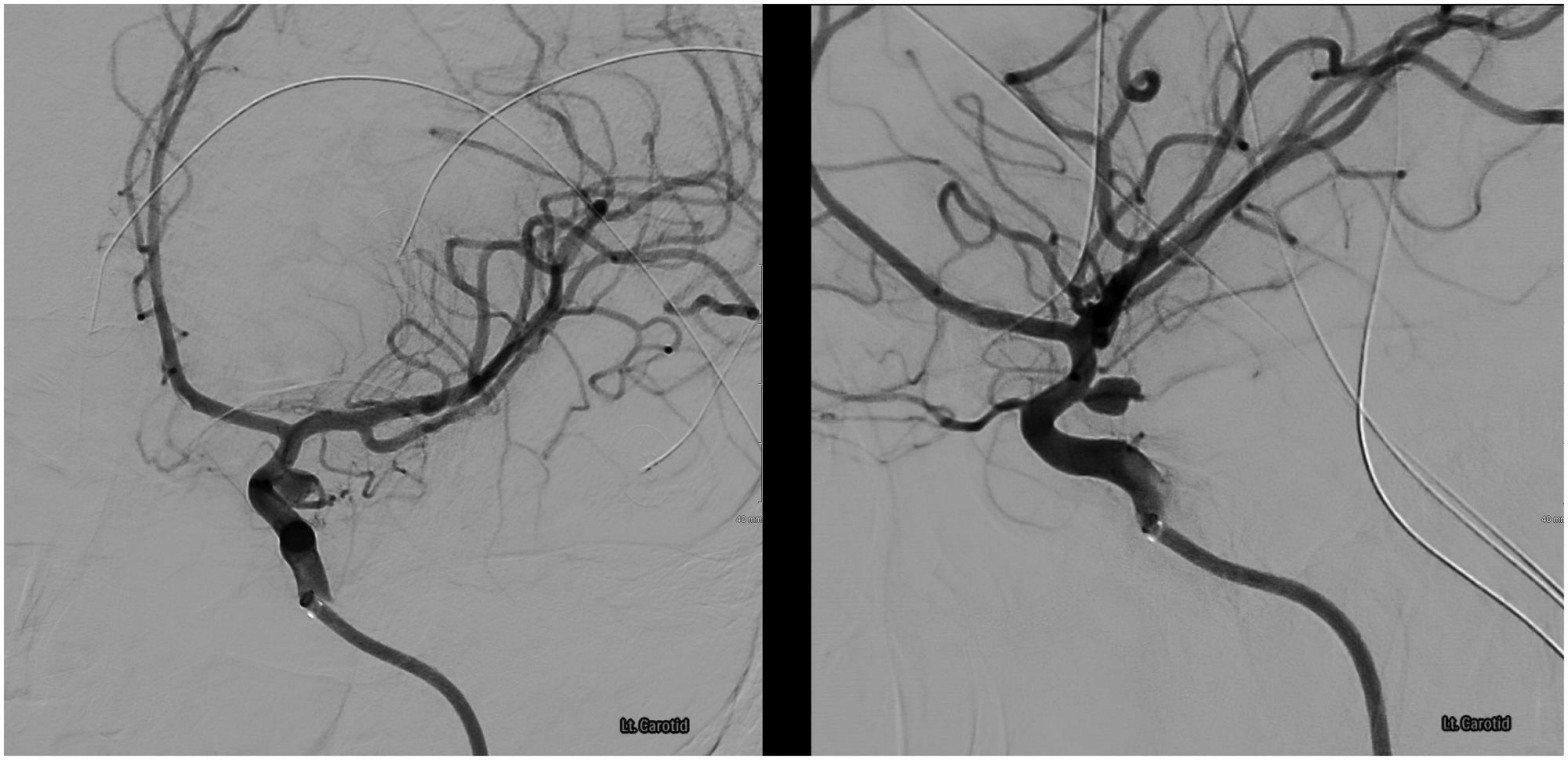
Pre-coiling angiography demonstrating a left posterior communicating segment internal carotid artery aneurysm.

The patient experienced painless uterine tightening suggestive of early stages of labour, and a vaginal examination by a consultant obstetrician was performed 12 hours post-admission, which confirmed that the cervix was closed. As mild uterine activity was noted (2 in 10 minutes), cardiotocography was used to track foetal well-being prior to coiling. To avoid foetal irradiation, a right radial access approach was used instead of a femoral approach, and a 7-French (FR) sheath (Cordis, United States) was inserted and used to inject 5000 units of heparin and 300 mcg of GTN. Verapamil was omitted due to the potential side effects of reduced uterine blood supply and foetal hypoxia. An additional 9000 units of heparin were administered during the procedure, guided by activated clotting time (ACT) monitoring, with a target ACT of >200 s. Baseline ACT was 98 s, which increased to 209 s during the procedure. A 7-FR RIST (Medtronic, Ireland) was advanced into the left ICA to the cavernous segment over a 5.5F Sim Select/0.035 Terumo (Terumo, Belgium). 3D and 2D angiography via the left ICA confirmed a 4 × 6 × 3 mm aneurysm (Figures 2 and 3). A Sceptre XC 4 × 11/Synchro Soft (Microvention, United States) was advanced across the aneurysm neck. The aneurysm was catheterised with a Headway 17 soft/Traxcess 14 (Microvention, United States) and then embolised with 4 detachable coils (Stryker and Microvention, United States). A Hydrofill 3 mm × 6 cm helical, Target helical nano 1.5 mm × 2 cm, Target 360 ultra 2 mm × 6 cm and Target 360 nano 1.5 mm × 3 cm were used.

Throughout the procedure, an abdominal lead apron was used to protect the foetus from irradiation, and the use of contrast was minimised to 100 mL to reduce the risk of contrast toxicity to the mother and foetus.

**Figure 4:**
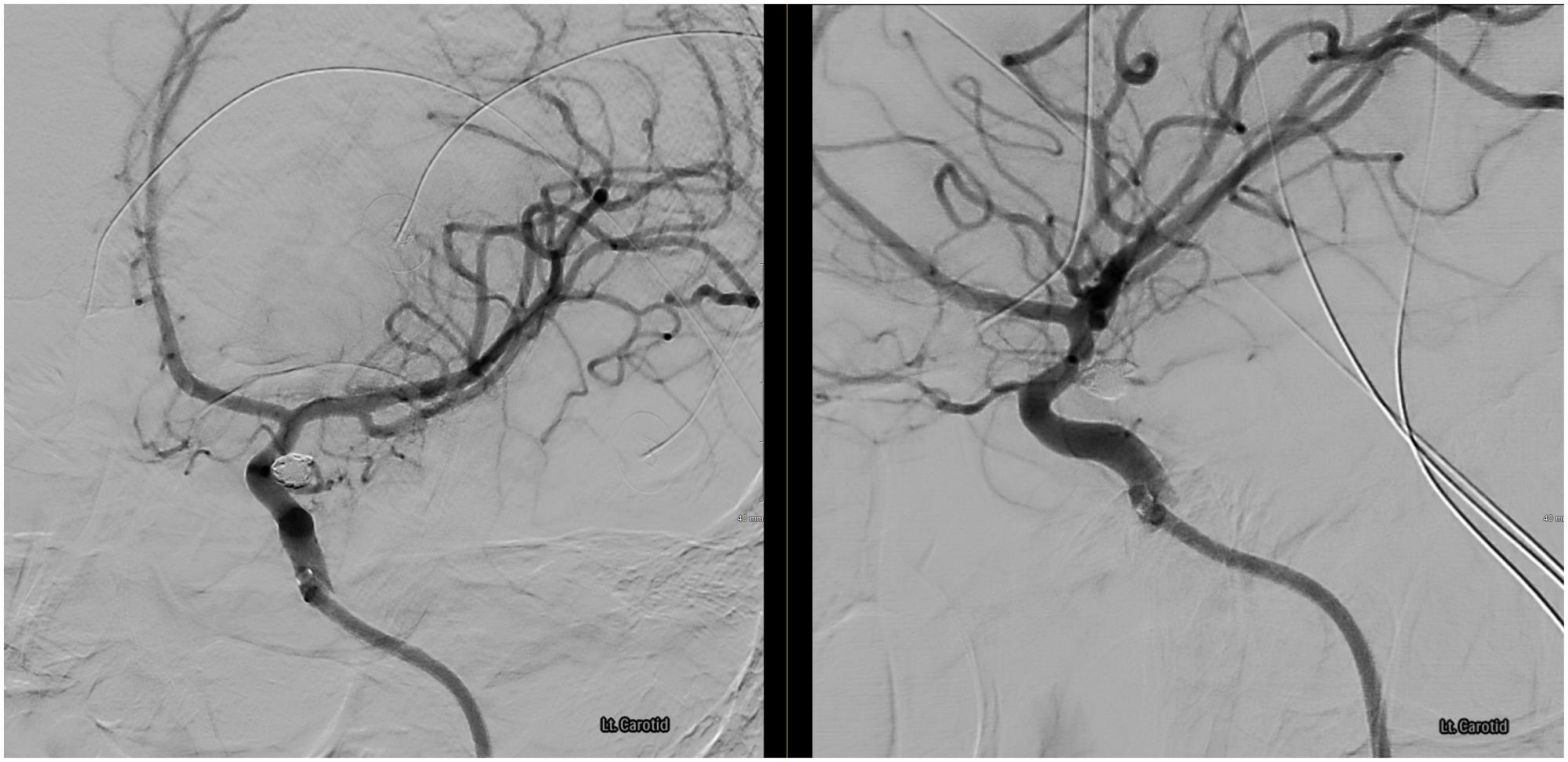
Post-coiling angiography demonstrating complete occlusion of the aneurysm.

Angiography confirmed modified Raymond Roy Class 1, complete occlusion of the aneurysm, with normal filling of the left anterior circulation (Figure 4). StatSeal (StatSeal, United States) and an inflation band were applied to the right radial artery puncture site. Following the procedure, the patient remained intubated and ventilated and was transferred immediately to theatres where foetal monitoring via CTG was commenced. One hour was allowed for the reversal of heparin, followed by a rapid entry lower-segment CS, which was uneventful. She was extubated postoperatively and then transferred to the neuro-critical care unit for monitoring.

## Follow-up

Following delivery, the patient was hypertensive (BP 170/104 mmHg), and labetalol and magnesium sulfate were administered as per the pre-eclampsia protocol. She was also on prophylactic aspirin and nimodipine. Over the next 2 days, the patient reported a headache radiating to her neck and back. CT head on day 2 post-op (with the day of the coiling and delivery as day 0) showed a trace of bilateral intraventricular haemorrhage in the occipital horns of the lateral ventricles. She no longer had hydrocephalus.

The headache became worse with standing and moving, and the patient suffered 30 minutes of anomic aphasia 6 days post-op. A CT brain perfusion study on day 6 post-op showed delayed temporal parameters in the left MCA territory, suggestive of vasospasm. Mean arterial pressure (MAP) was then augmented to 120 mmHg, yet she suffered another episode of expressive dysphasia and right pronator drift on day 7 post-op. MAP was augmented to 130 mmHg on day 7 post-op, which resulted in an improvement of her speech; however, a mild pronator drift persisted.

CTP/CTA on day 7 post-op revealed significant narrowing in the distal left internal carotid artery (ICA), A1, and M1 segments, with collateral blood flow supplying the left anterior cerebral artery (ACA) territory. There was prolongation of the temporal parameters throughout the left middle cerebral artery (MCA) territory without evidence of infarction. Digital subtraction angiography with intra-arterial verapamil (10 mg) in the left terminal ICA was administered, resulting in improvement in the appearances of the terminal ICA and M1 spasm, with filling of the left A1.

## Review of literature

**Figure 5:**
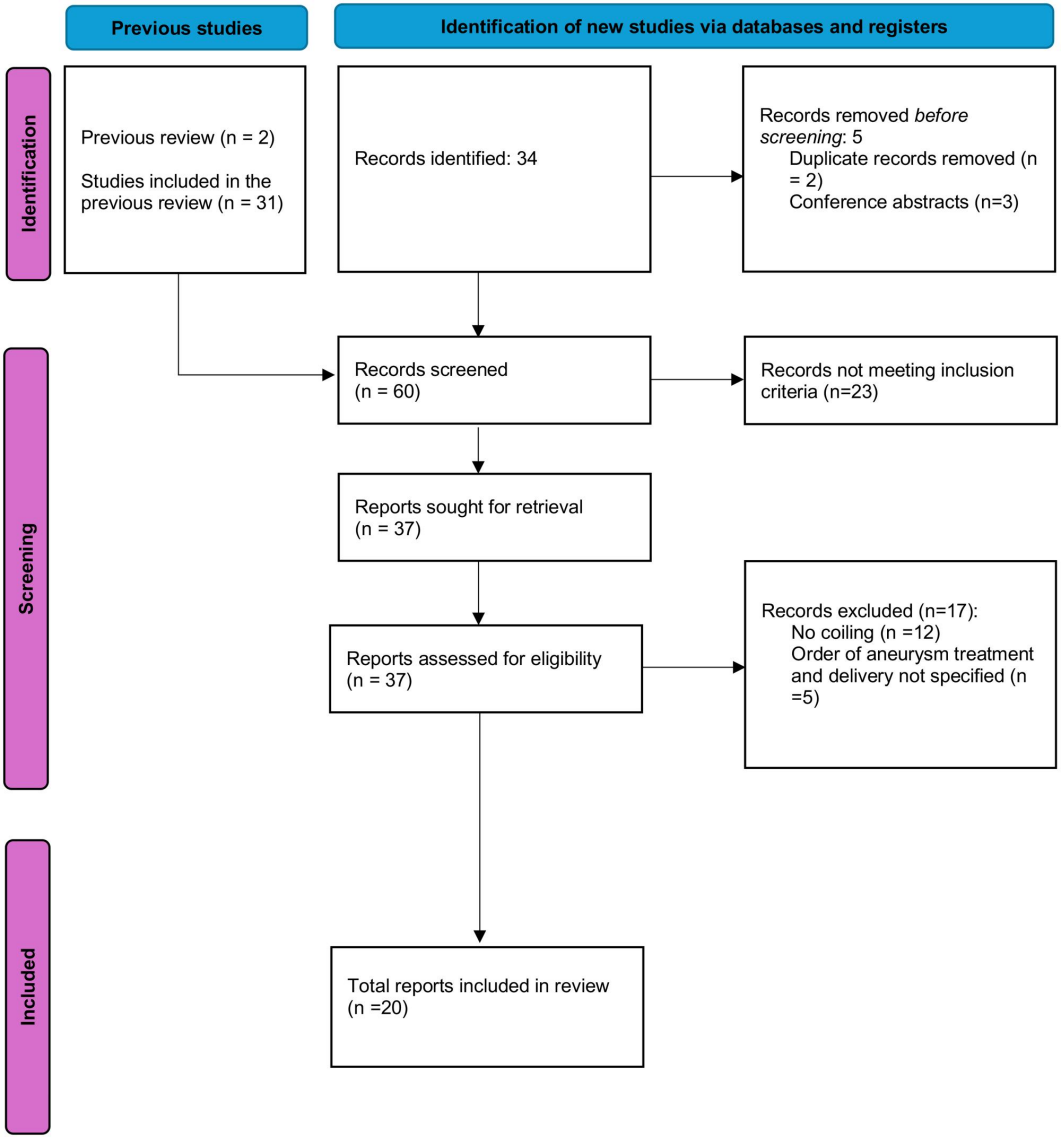
Study selection process for literature review.

We evaluated papers from 2 systematic reviews: Nussbaum et al.^6^ and Beighley et al.,^7^ and performed an updated literature search. Unlike previous reviews, our work focused on the management of ruptured IAs at term and the sequence of treatment. We used papers identified in the above systematic reviews and conducted an updated search of PubMed and ClinicalTrials.gov with the search terms “Intracranial Aneurysm” AND “Pregnancy” for full-text papers from January 2019 onwards. We did not exclude case reports. This search yielded 34 results. Figure 5 shows the study selection process.

### Inclusion criteria

- Case reports or records that document at least 1 case of ruptured intracranial aneurysm.
- The aneurysm was treated via endovascular coiling.
- The treatment occurred during pregnancy or the peripartum period.
- The case included clear information on gestational age or timing of coiling and delivery.
- The report provided details on maternal and foetal outcomes.

### Exclusion criteria

- Cases involving unruptured intracranial aneurysms.
- Cases involving arteriovenous malformations (AVMs).
- Cases where aneurysms were treated via non-coiling methods (e.g., surgical clipping).
- Cases where treatment occurred outside of pregnancy or the peripartum period.
- Records that lacked sufficient clinical detail regarding the timing of intervention or maternal/foetal outcomes.

## Results of literature review

We identified 29 cases of ruptured intracranial aneurysms in pregnancy where the order of coiling and CS was clearly described. All 9 patients presenting at <22 weeks of gestation underwent coiling followed by interim delivery, which is delivery that takes place after foetal maturity, balancing the risks of prematurity with ongoing maternal neurovascular support. 20 cases presented after 22 weeks; 11 of whom underwent coiling first. Women who underwent coiling before delivery had an average gestational age of 30.3 weeks at the time of the coiling procedure and delivered at an average of 36.1 weeks of gestation. Women who delivered prior to coiling underwent the coiling procedure and delivered at approximately the same gestational age, with an average of 35.1 weeks for both events. Three out of 9 women who delivered first died. One of the 10 women who were coiled first died.

## Key issues/discussion

Aneurysmal subarachnoid haemorrhage is one of the non-obstetric causes of maternal deaths during pregnancy, and most of these ruptures occur in the third trimester.^8^ There are currently no treatment guidelines, and treatment decisions are individualised according to the aneurysm location, morphology, size, and patient parameters.^6^ To our knowledge, this case represents the latest gestation (39 + 3 weeks in the prelabour phase) in which a ruptured intracranial aneurysm was secured before delivery of the baby in the pre-labour phase.

Previous work on this topic^9^ has sought to describe the differences in outcomes with various treatment decisions. Our study expands on this by offering a data-driven comparison of maternal and foetal outcomes based on the sequence of aneurysm treatment and delivery, focusing exclusively on endovascular cases.

## Early pregnancy

For pregnancies under 22 weeks’ gestation, the foetus is generally considered to be non-viable.^10^  Table 1 shows that all cases (*n* = 9/9) in this gestation category underwent coiling first, followed by interim delivery. This supports the approach that maternal stabilisation and aneurysm securing should take precedence during early pregnancy.

**Table 1:** Cases where the patient presented under 22 weeks of gestation. All patients underwent coiling first.

First author & Year	Gestation at coiling (weeks)	Gestation at delivery (weeks)	Location of aneurysm	Stent/balloon	Foetal outcome	Maternal outcomes	Delivery Mode
Ueda, 2020^24^	8	36	ICA-Pcomm junction	No	Healthy	Healthy	CS
Kizilkilic, 2003^25^	10	NA	Pcomm	Balloon	Aborted	Healthy	NA
Kizilkilic, 2003^25^	18	35	ICA	Balloon	Healthy	Healthy	CS
Deshmukh 2023^26^	12	39	ICA 4	Flow diverter	Healthy	Healthy	CS
Kim, 2014^27^	16	38	PICA	None	Healthy	Healthy	CS
Pop R, 2020^28^	17	39	Ophthalmic segment of ICA	Balloon then flow diverter	Healthy	Healthy	Vaginal
Piotin, 2001^29^	20	40	ICA bifurcation	None	Healthy	Third nerve palsy	CS
Liu, 2015^5^	20	38	Pcomm segment of ICA	None	Healthy	Healthy	CS
Kasashima, 2024^21^	21	37	Pcomm	Balloon	Healthy	Healthy	CS

Abbreviations: CN III palsy = cranial nerve III (oculomotor) palsy; ICA = internal carotid artery; NA = not available or not applicable; Pcomm = posterior communicating artery; PICA = posterior inferior cerebellar artery. “Stent/balloon” indicates use of adjunctive devices, including balloon assistance or flow diverters.

## Late pregnancy

Patients presenting later than 22 weeks’ gestation underwent either coiling or delivery first (Table 2). The average maternal gestation undergoing coiling first (*n* = 11/20) was 30.3 weeks, whilst patients undergoing delivery first (*n* = 9/20) had an average gestation of 35.1 weeks. This suggests that, in the cases identified in our literature review, delivering the foetus was preferred before coiling if the foetus was viable and closer to term.

**Table 2:** Cases where the patient presented greater than 22 weeks of gestation.

First author & Year	Gestation at coiling (weeks)	Gestation at delivery (weeks)	Location	Coiling first	Delivery first	Stent/balloon	Foetal outcome	Maternal outcomes	Delivery Mode
Liu, 2015^5^	26	NA	Basilar tip	✔		Stent	Died in utero	Dead	NA
Karabuk, 2020^30^	27	35	Acomm	✔		No	Healthy	Healthy	CS
Surico, 2015^31^	27	27	R ICA	✔		No	Healthy	Healthy	CS
Kizilkilic, 2003^25^	28	38	Accom	✔		No	Healthy	Healthy	CS
Tarnaris, 2012^32^	29	38	Pcomm	✔		No	Healthy	Third nerve palsy	CS
Vatsa, 2019^33^	28 and 36	36	PCA	✔		No	Healthy	Healthy	CS
Oi, 2022^34^	30	38	A1 segment of the ACA	✔		No	Healthy	Healthy	CS
Xie, 2021^1^	32	37	Ophthalmic segment of L ICA	✔		No	2x Healthy	Healthy	CS
Piotin, 2001^29^	32	32	ICA		✔	No	Healthy	Healthy	CS
Pumar, 2009^35^	32	38	Basilar	✔		No	Healthy	Healthy	CS
Meyers, 2000^36^	33^a^	38	Basilar	✔		No	Healthy	Healthy	CS
Meyers, 2000^36^	37^b^	37	Pcomm		✔	No	2x Healthy	Healthy	CS
Roman, 2004^17^	35	35	Carotid opthalmic		✔	No	Healthy	Healthy	CS
Riviello, 2004^37^	36	36	ACA pericallosal		✔	No	Healthy	Healthy	CS
Liu, 2015^5^	36	36	Pcomm		✔	No	Healthy	Dead	CS
Guida, 2012^38^	37	37	MCA bifurcation		✔	No	NA	Dead	CS
Guida, 2012^38^	32	32	Acomm		✔	No	Healthy	Dead	CS
Guida, 2012^38^	33	33	Basilar tip		✔	No	Healthy	CN III palsy	NA
Shahabi, 2001^39^	38	38	Basilar tip		✔	No	Healthy	Healthy	CS

a Reported as mid third trimester.

b Reported as late third trimester.

ACA = anterior cerebral artery; AComm = anterior communicating artery; ICA = internal carotid artery; MCA = middle cerebral artery; PCA = posterior cerebral artery; PComm = posterior communicating artery; R/L ICA = right/left ICA.

✔ in “Coiling first” or “Delivery first” indicates which occurred first. “Stent/balloon” notes use of adjunctive devices. CS = Caesarean section. CN III palsy = cranial nerve III (oculomotor) palsy. NA = not available or not applicable. Twin pregnancies or repeated procedures are noted where relevant.

All cases of maternal mortality (*n* = 3) occurred in cases presenting at more than 36 weeks of gestation and in those who underwent delivery of the baby first. This may be related to haemodynamic changes near term, specifically increased heart rate, cardiac output and blood pressure, and the further increase in cardiac output in the first hour after delivery,^11^ resulting in an increased risk of aneurysm re-bleed.

## Impact of the sequence of management on maternal and foetal outcomes

**Figure 6:**
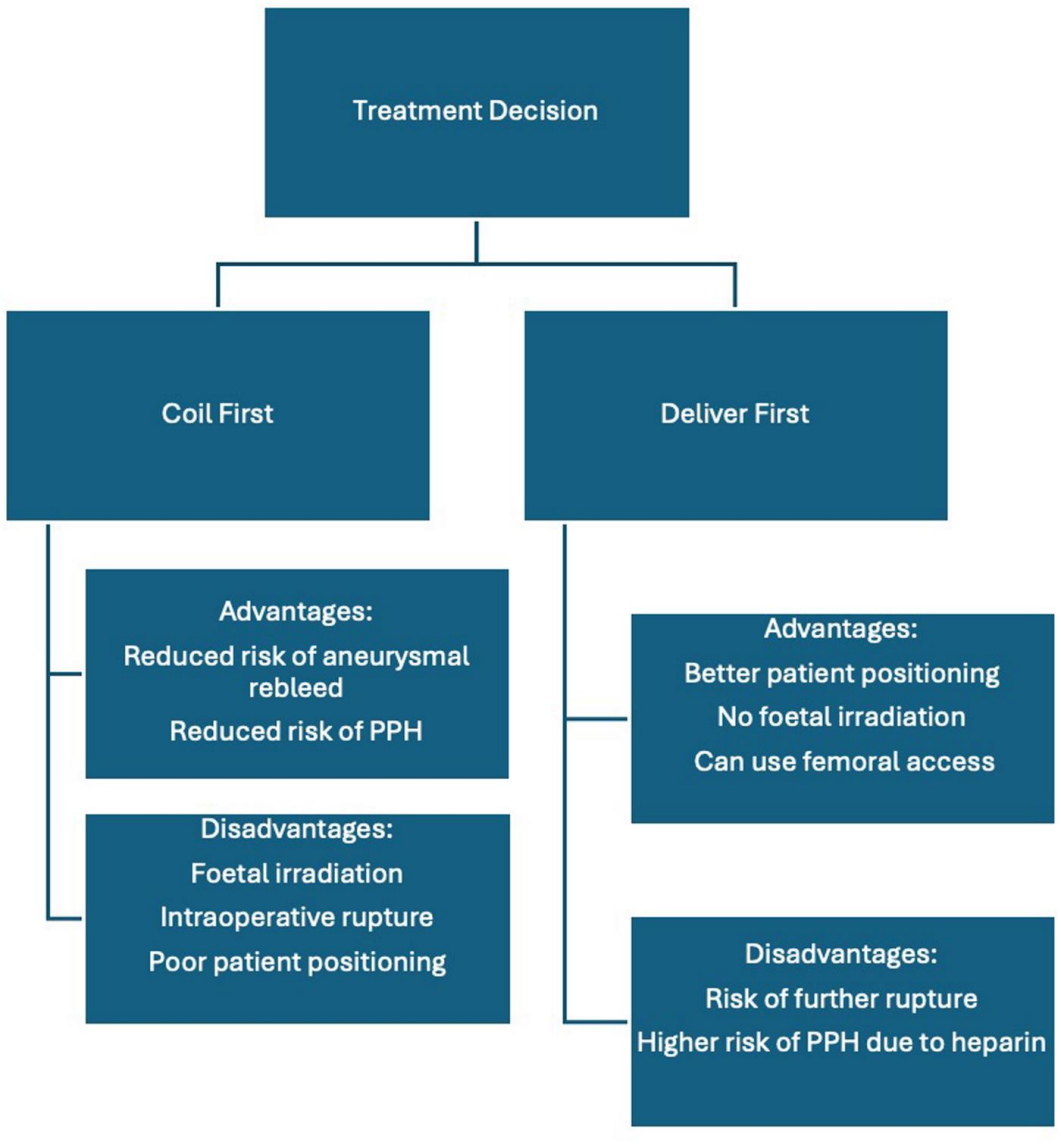
Summary flow chart of the pros and cons of coiling versus delivering the foetus first. PPH—postpartum haemorrhage.

In the absence of obstetric concerns, ruptured aneurysms should be treated with the same urgency and methods as non-pregnant patients, with necessary modifications to protect the foetus.^12^ However, a multidisciplinary approach is necessary when the pregnant patient is at term or in the prelabour phase.^13^  Figure 6 summarises the major factors that should be considered when deciding the order of delivery and treatment. Patients should be made aware of the risks and benefits of both approaches and be involved in the decision-making process.

Delivering the foetus first carries risk of aneurysm re-rupture and re-bleeding due to haemodynamic changes.^4^ Pooled data analyses of 44 cases suggests that women who underwent aneurysm coiling before or during the same admission as delivery had better functional outcomes.^14^ An argument could be made that the use of systemic heparin during coiling may increase the risk of bleeding in a subsequent CS due to residual heparin. However, heparin reversal with protamine sulfate and activated clotting time monitoring can mitigate such bleeds. Where possible, allowing an interim period between coiling and delivery may reduce the risk of postpartum haemorrhage as the remaining heparin is excreted.^15^

Delivering first has been proposed to result in a better outcome for the foetus^16^^,^^17^ as it is protected from anaesthesia-related complications and spared the changes in maternal blood pressure dynamics that could occur during the procedure. However, in most cases, the risks of general anaesthetic to the foetus are low and minimising the procedure time can reduce this risk further.^18^

There is also a risk of foetal irradiation during coiling, which can be minimised with adequate lead shielding and optimisation of fluoroscopy techniques. Typical foetal radiation dose during endovascular coiling ranges from 0.17 to 2.8 mGy,^19^ which is well below the 50-100 mGy range associated with adverse effects.^20^ Using a transradial approach can further reduce this radiation risk to the foetus.^21^

## Mode of delivery

There is a strong consensus in the literature that vaginal delivery is not appropriate in patients with unsecured intracranial aneurysms.^4^ There are several factors which influence this decision. The direct Valsalva manoeuvres exerted in labour may increase the risk of rupture in untreated aneurysms. Some studies found there was a greater incidence of rupture of untreated and unruptured aneurysms during vaginal delivery compared to CS.^4^ However, other studies^22^^,^^23^ found that the risk of intracranial haemorrhage in the presence of an untreated cerebrovascular lesion was not significantly different between vaginal delivery and CS. Once the aneurysm is secured, there is no difference in risk of re-bleed between CS and vaginal delivery.^4^

Given the rarity of ruptured intracranial aneurysms presenting during pregnancy and labour, the available evidence is largely limited to case reports and small series. A consistent theme emerging from the literature is the prioritisation of maternal aneurysm treatment over foetal delivery, and a focused appraisal of the optimal sequence of intervention is therefore clinically relevant. Across reported cases, maternal mortality was higher among patients who underwent delivery prior to aneurysm treatment (3/9) compared with those who underwent aneurysm coiling before delivery (1/11). Notably, all reported maternal deaths (*n* = 3) which occurred in patients presenting beyond 36 weeks’ gestation underwent delivery prior to aneurysm treatment. This observation is discussed in the context of established physiological changes near term, including increases in heart rate, cardiac output, and blood pressure, which may increase the risk of aneurysm re-bleeding. Given that ruptured aneurysms require treatment within 24 hours to minimise re-bleeding risk, the time-critical nature of management makes decisions regarding treatment sequence particularly challenging. We believe this work provides practical guidance for neurointerventionalists managing these rare but high-risk scenarios.

## Conclusion

Our case demonstrates favourable maternal and foetal outcomes following endovascular coiling of a ruptured aneurysm prior to delivery in a pregnant patient presenting during labour. Our review of the available literature supports securing the ruptured aneurysm before delivery where feasible, to minimise the risk of re-bleeding. Maternal mortality appears higher in cases where delivery was prioritised over aneurysm treatment, underscoring the potential importance of aneurysm securing prior to delivery. Further prospective, registry-based studies and comprehensive long-term follow-up are required.

## Learning points

- Subarachnoid haemorrhage from ruptured intracranial aneurysms in pregnancy is rare, with no clear treatment guidelines.
- Case at term: coiling followed by caesarean section resulted in good maternal and foetal outcomes.
- Literature review suggests securing the aneurysm before delivery reduces mortality.
